# A longitudinal study on SARS-CoV-2 seroconversion, reinfection and neutralisation spanning several variant waves and vaccination campaigns, Heinsberg, Germany, April 2020 to November 2022

**DOI:** 10.2807/1560-7917.ES.2024.29.26.2300659

**Published:** 2024-06-27

**Authors:** Bianca Schulte, Enrico Richter, Antonia Büning, Maximilian Baum, Annika Breuer, Jasmin Zorn, Julia König, Melanie Geiger, Monika Eschbach-Bludau, Johanna Heuser, Dominik Zölzer, Marek Korencak, Ronja Hollstein, Eva Beins, Dorian Emmert, Souhaib Aldabbagh, Anna Maria Eis-Hübinger, Hendrik Streeck

**Affiliations:** 1 Institute of Virology, University Hospital Bonn, Venusberg-Campus 1, Bonn, Germany; 2 German Center for Infection Research (DZIF), Partner Site Bonn-Cologne, Bonn, Germany; 3 Institute of Human Genetics, School of Medicine and University Hospital Bonn, Venusberg-Campus 1, Bonn, Germany

**Keywords:** SARS-CoV-2, COVID-19, pandemic preparedness, Omicron, BA.1, BA.2, BA.5, XBB1.5, BQ.1.1, cohort study, seroconversion, long-term immunity

## Abstract

**Background:**

Since its emergence in December 2019, over 700 million people worldwide have been infected with SARS-CoV-2 up to May 2024. While early rollout of mRNA vaccines against COVID-19 has saved many lives, there was increasing immune escape of new virus variants. Longitudinal monitoring of population-wide SARS-CoV-2 antibody responses from regular sample collection irrespective of symptoms provides representative data on infection and seroconversion/seroreversion rates.

**Aim:**

To examine adaptive and cellular immune responses of a German SARS-CoV-2 outbreak cohort through several waves of infection with different virus variants.

**Methods:**

Utilising a 31-month longitudinal seroepidemiological study (n = 1,446; mean age: 50 years, range: 2–103) initiated during the first SARS-CoV-2 superspreading event (February 2020) in Heinsberg, Germany, we analysed acute infection, seroconversion and virus neutralisation at five follow-up visits between October 2020 and November 2022; cellular and cross-protective immunity against SARS-CoV-2 Omicron variants were also examined.

**Results:**

SARS-CoV-2 spike (S)-specific IgAs decreased shortly after infection, while IgGs remained stable. Both increased significantly after vaccination. We predict an 18-month half-life of S IgGs upon infection. Nucleocapsid (N)-specific responses declined over 12 months post-infection but increased (p < 0.0001) during Omicron. Frequencies of SARS-CoV-2-specific TNF-alpha^+^/IFN-gamma^+^ CD4^+^  T-cells declined over 12 months after infection (p < 0.01). SARS-CoV-2 S antibodies and neutralisation titres were highest in triple-vaccinated participants infected between April 2021 and November 2022 compared with infections between April 2020 and January 2021. Cross neutralisation against Omicron BQ.1.18 and XBB.1.5 was very low in all groups.

**Conclusion:**

Infection and/or vaccination did not provide the population with cross-protection against Omicron variants.

**Key public health message**

**What did you want to address in this study and why?**

We wanted to better understand the long-term immune responses against SARS-CoV-2 in the population, and how they were influenced by the COVID-19 vaccination campaigns and the emergence of new SARS-CoV-2 variants. We studied a small German community over 31 months, which had experienced a large COVID-19 outbreak during the early pandemic in Germany.

**What have we learnt from this study?**

We estimated the rate at which immunity against the virus fades in the population. Our study also demonstrated the consequences of SARS-CoV-2´s changeability, by showing that neither three vaccinations nor infections with earlier variants, or a combination of both, protects from infection with the more recent Omicron variants of the virus.

**What are the implications of your findings for public health?**

The ability to follow a community throughout the pandemic to monitor their immune responses can help public health authorities to formulate guidelines on vaccination, testing and hygiene measures in times of infectious disease outbreaks.

## Introduction

Only one year after the beginning of the COVID-19 pandemic, the first mRNA vaccine was approved for use in humans in December 2020. Thus, the spread of severe acute respiratory syndrome coronavirus 2 (SARS-CoV-2) was likely altered by the presence of vaccine-induced antibodies in most populations [1]. In addition to vaccines, other public health measures, e.g. lockdowns, masking and social distancing, also hindered transmission over the course of the pandemic. 

Longitudinal monitoring of antibody responses in the population can help to characterise the longevity of protection after both infection and vaccination, or a combination thereof. In the beginning of the pandemic, seroconversion rates were high (up to 90.7%) upon infection with the ancestral SARS-CoV-2 strain [2], while the average seroreversion time was 24 months [3] and immune responses produced robust neutralising antibody titres [4,5]. SARS-CoV-2 spike (S) protein-specific immune responses were triggered by both infection and vaccination, since early COVID-19 vaccines were designed to target S antigens. In contrast, antibodies against the nucleocapsid (N) antigen were triggered exclusively during infection before the first N-specific vaccine was tested in human trials (February 2023) [6], making it a valuable tool for tracking infection rates in both unvaccinated and vaccinated populations. 

The extent to which antibody responses correlate with virus neutralisation and immune protection remains a subject of ongoing debate. It has been demonstrated that T-cell responses against SARS-CoV-2 exhibit greater longevity compared with neutralising antibodies, indicating the pivotal role played by cellular immunity [4]. The analysis of SARS-CoV-2-specific immune responses was further complicated by the subsequent emergence of new virus variants, especially Omicron, which arose in November 2021, which showed a different antigenic profile from the ancestral strain [7]. It became evident that it had accumulated > 50 mutations in comparison to ancestral virus, 30 of which lay within the S gene (15 in the receptor binding domain (RBD) alone) [8]. Importantly, at the time the Omicron variant emerged, the majority of the world’s population had not developed hybrid immunity, i.e. one or more infections followed by vaccination, or vice versa. A strong diversification of Omicron followed, resulting in dozens of subvariants, such as BA.1, BA.2, and BA.5, BQ.1.18 and XBB.1.5, the latter of which resulted from recombination of variants BJ.1 and BM.1.1.1 [9]. Some of these variants show very different antigenic profiles and levels of immune escape [10].

Under these complex circumstances, long-term surveillance of epidemiological trends and in-depth exploration of the nature and scope of immune responses can provide invaluable insights for pandemic preparedness and the design of future vaccines. In February 2020, a large COVID-19 outbreak took place at a festival in Heinsberg, Germany [5,11]. Using an established cohort of volunteers that included festival participants and town inhabitants, we examined the SARS-CoV-2-specific immune responses over a 31-month time span from April 2020 to November 2022. 

## Methods

### Study setting, participants and sample collection

A longitudinal SARS-CoV-2 seroepidemiological cohort study was performed using an established cohort (n = 1,446) from a cross-sectional study of the first German COVID-19 superspreading event (festival) in Gangelt (municipality of Heinsberg) Germany (population of 13,240 on 31 Dec 2022) in February 2020 [5,12]. This cross-sectional study constitutes the baseline for our present analysis. 

Inclusion criteria for the follow-up study presented here were residence in the town at the time of the superspreading event, or participation in the event. All participant data were entered twice independently into a RedCap database and the two inputs crosschecked for discrepancies.

Participants were invited to five subsequent visits for sample collection: Visit 1 (month 6, October 2020), Visit 2 (month 9, January 2021), Visit 3 (month 12, April 2021), Visit 4 (month 26, June 2022) and (month 31, November 2022). The minimum number of visits for inclusion was one. New volunteers were accepted up to Visit 4. Supplementary Figure S1A provides an overview over enrolment and testing numbers.

Participants were asked to donate blood, pharyngeal swab and saliva at each visit. The sampling protocol for our cohort was established by our research team prior to the initial visit (April 2020, Visit 0) and remained consistent throughout the study’s duration. This consistency ensured that the positivity rate reported in our cohort reflects a standardised approach to testing within our specific research context. Participants who preferred incomplete sample donation (blood, pharyngeal swab or saliva only, or a combination of two) were also included in the study. The blood was processed for serology and flow cytometry, and the pharyngeal swab was processed for RT-PCR and/or next generation sequencing (NGS) (described below). Saliva was stored at −20 °C for further analyses that were not included in this manuscript. qPCR was performed by the virology diagnostics department of the University Hospital Bonn.

Rollout of COVID-19 mRNA vaccine Comirnaty (BNT162b2 mRNA, BioNTech-Pfizer) in Germany started at the end of December 2020, followed by the Vaxzevria DNA vaccine (ChAdOx1 nCoV-19, Oxford-AstraZeneca) and the Spikevax mRNA vaccine (mRNA-1273, Moderna) in January 2021. Participants included in our study were vaccinated 0–5 times throughout the study period between January 2021 and November 2022. Data on vaccination status was collected via self-administered questionnaire. Those participants grouped for analysis of Visit 4 (June 2022) were vaccinated between January 2021 and April 2022.

### Sample processing and analysis

#### SARS-CoV-2 serology

Plasma from venous blood was aliquoted, stored at −80 °C or stored at 4 °C overnight for ELISA testing. Euroimmun SARS-CoV-2 IgA and IgG ELISAs (S1 domain-specific) and Roche Elecsys anti-SARS-CoV-2 ELISA (nuclecapsid protein-specific, detects IgA, IgG and IgM) were performed.

#### Peripheral blood mononuclear cell isolation and flow cytometry

Peripheral blood mononuclear cell (PBMC) isolation was performed via Ficoll gradient centrifugation, as described earlier [13]. Cryopreserved PBMCs were thawed and allowed to rest overnight, stimulated with wild-type SARS-CoV-2 PepTivator overlapping peptide pools (Miltenyi Biotec) of S, N or membrane (M) proteins. Cells (1x10^6^) were stimulated for 6 h with BD FastImmune co-Stimulatory antibodies (CD28/CD49d) and anti-CD107a-Pe/Dazzle (Biolegend). GolgiStop and GolgiPlug (BD Bioscience) was added 1 h into the stimulation. Cells were stained with ZombieAqua (Biolegend), washed with FACS buffer, stained for extracellular (anti-CD3-APC-Cy7, clone UCHT1, Biolegend; anti-CD4-BV786, clone SK3, BD Bioscience; anti-CD8-AF700, clone RPA-T8, Biolegend) and intracellular markers (anti-IL-2-FITC, clone MQ1-17H12, Biolegend; anti-IFN-gamma-PE, clone B27, Biolegend; anti-TNF-alpha-BV605, clone Mab11, Biolegend; anti-CD107a-PE/Dazzle 594, clone H4A3, Biolegend), and acquired on FACS Celesta (BD Bioscience). Data were analysed with FlowJo Software version 10.0.7 (TreeStar).

#### Neutralisation assays

Neutralisation assays were performed with IgG-positive plasma samples as previously described [12]. Briefly, collected plasma was heat-inactivated, incubated with ancestral/variant SARS-CoV-2 for 1 h at 37 °C, then used to infect Vero E6 cells. The virus–plasma mix was replaced by overlay media (0.75% (w/v) carboxymethylcellulose (Sigma) in MEM + 2% (v/v) FBS + 0.22% (w/v) NaHCO_3_ + penicillin/streptomycin), incubated for 72 h, then cells were fixed, stained with crystal violet and plaques counted.

A large batch of each virus stock was produced by propagation first on Caco-2 cells (passage 1) and then on Vero E6 cells (passage 2). Passage 2 was cleared of debris by centrifugation (3,200 g for 10 min), sterile filtered (0.2 μm), aliquoted and stored at −80 °C until use. One aliquot was submitted to NGS, and for the data presented here, the same virus batch was used.

#### SARS-CoV-2 RT-PCR

SARS-CoV-2 RT-PCR for both S and envelope (E) genes was performed as previously described [5]. Briefly, viral RNA was extracted using either the Chemagic Prime instrument platform (IVD-1033-S chemagic Viral DNA/RNA 300 Kit H96 (Revvity, Inc.) or the AltoStar Purification kit 1.5 RUO on the AltoStar AM16 automated pipetting platform (altona Diagnostics). RT-PCR was performed either with the SARS-CoV-2 RT-PCR kit 1.5 (altona Diagnostics) or swab samples were extracted and analysed via the Alinity m platform (Abbott). All assays were performed according to manufacturer’s instructions. Quantification cycle (Cq) values were compared with an internal control.

#### Next generation sequencing 

Next generation sequencing was performed on SARS-CoV-2 propagated from pharyngeal swabs of SARS-CoV-2 RT-PCR-positive volunteers as previously described [11]. Kits used were the NEBNext Artic Sars-CoV-2 FS Library Prep Kit (E7658L) from New England Biolabs (NEB) using the VarSkip short v2 primer for optimal coverage of variants, NEBNext Multiplex Oligos for Illumina (E6440S), NEBNext Sample Purification Beads (NEBNext Ultra II kit), Qubit dsDNA HS (High Sensitivity) Assay Kit (Thermo Fisher), D1000 Screen Tapes (Agilent Technologies), and the MiSeq Reagent Micro Kit V2 or MiSeq Reagent Kit V2 (Illumina). For sequence analysis, CLC Genomic Workbench version 23.0.2 (Qiagen) and NextClade Web (version 1.5.4) were used.

### Statistical analysis

Statistical analyses were performed with GraphPad Prism 9.5.1 or R version 4.2.3 (R Foundation, Vienna, Austria). Kruskal-Wallis and Wilcoxon signed-rank test were used for nonparametric analysis. P values < 0.05 (*), < 0.01 (**), < 0.001 (***), or < 0.0001 (****) were considered to be significant. Neutralisation capacities in different age groups were compared by unpaired t tests.

## Results

### Characterisation of the study cohort

The total number of participants for the follow-up seroprevalence analysis was 1,004, which included 726 for Visit 1, 1,004 for Visit 2, 883 for Visit 3, 597 for Visit 4 and 495 for Visit 5. The mean age per visit ranged from 47 to 54 years (range: 2–103 years). Supplementary Figure S1 provides an overview of the participant numbers, total and positive PCR tests and number of blood samples taken for all follow-up visits. The sex and age distribution of the cohort is provided in Supplementary Figure S2. 

### Longitudinal antibody responses during SARS-CoV-2 variant waves

**Figure 1:**
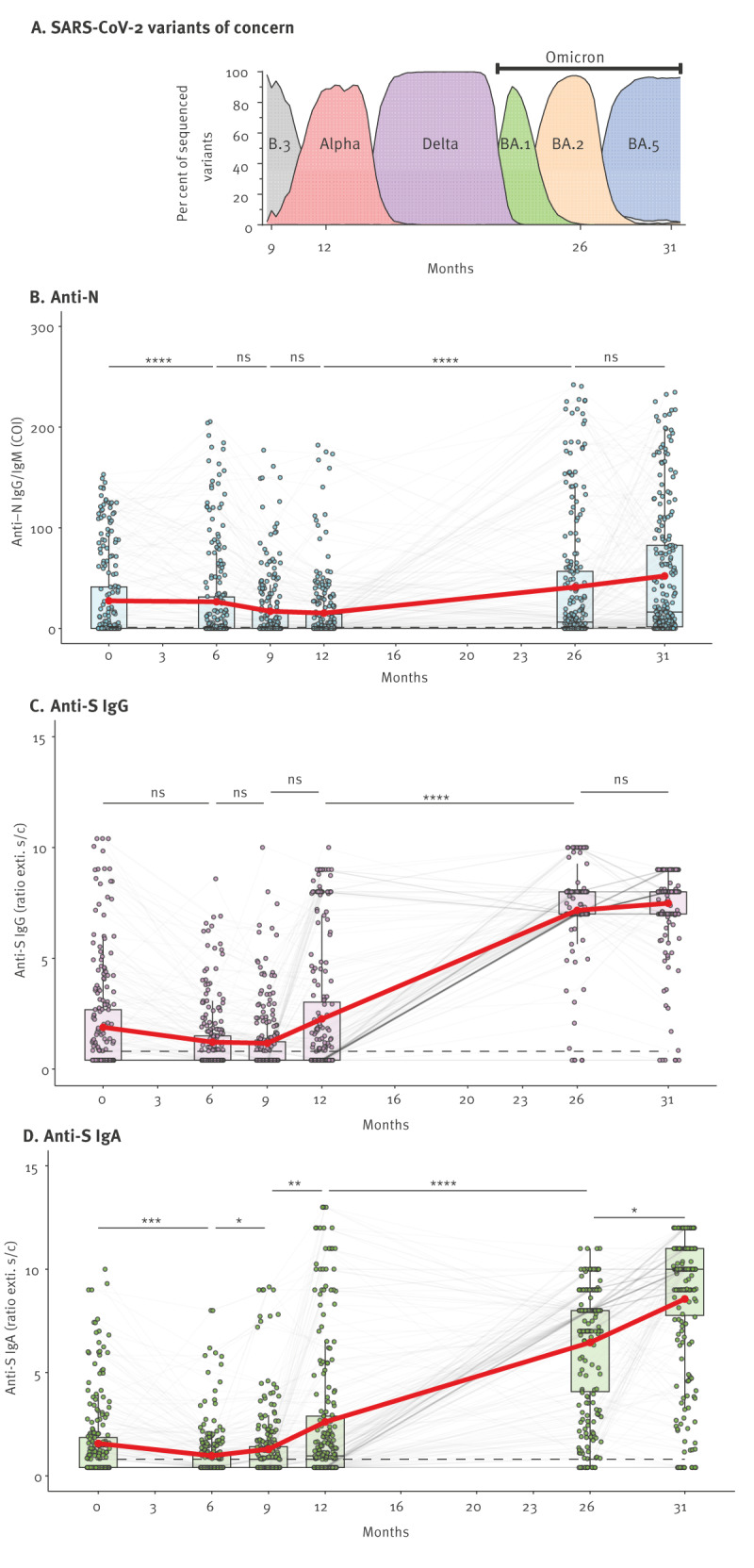
Waves of SARS-CoV-2 variants of concern and antibody responses in the study cohort at six time points, Heinsberg, Germany, April 2020–November 2022 (n = 231 individuals)

Throughout the successive waves of SARS-CoV-2 infections in Germany, five different variants of concern (VOC) emerged. These variants originated from the ancestral strain (B.3) and included the Alpha, Delta and Omicron VOCs BA.1, BA.2 and BA.5 (Figure 1A). Supplementary Figure S3 provides timelines of infection numbers in Germany vs the community from which our cohort originates. 

COI: cut-off index; exti. s/c: extinction of sample divided by extinction of calibrant; IgA: immunoglobulin A; IgG: immunoglobulin G; N: SARS-CoV-2 nucleocapsid protein; ns: not significant; S: SARS-CoV-2 spike protein; SARS-CoV-2: severe acute respiratory syndrome coronavirus 2.

A. Waves of SARS-CoV-2 variants of concern in Germany between Visit 2, January 2021 and Visit 6, November 2022 [11]. These data are based on a nationwide next generation sequencing (NGS) screening database collected from all German hospitals with NGS capacity (Robert Koch Institute national surveillance database). Nationwide sequencing was available from January 2021. Note that the SARS-CoV-2 mRNA vaccine rollout in Germany started in December 2020, i.e. shortly before month 9 of this study.

B–D. Longitudinal antibody responses of volunteers who participated in all visits (n = 231) shown as total N, S IgG and S IgA. Data are represented as individual points and box plots (interquartile range (IQR) with first quartile (Q1), median and third quartile (Q3). Minimum is defined as Q1−1.5xIQR and maximum is defined as Q3+1.5xIQR). The red line connects the median values of each time point and the dashed line represents the positivity cut-off. Differences between the time points were assessed by the Wilcoxon signed-rank test. P values < 0.05 (*), < 0.01 (**), < 0.001 (***), or < 0.0001 (****) were considered to be significant.

When comparing antibody responses during the different VOC waves from 231 study volunteers who participated in all study time points, we observed that N-specific responses (Figure 1B) remained stable for the first 6 months after infection, which were dominated by the B.3 variant. Subsequently, N-specific antibodies declined and reached their lowest levels by month 12, then significantly resurged at month 26 (June 2022) during the Omicron BA.2 wave. The sample size and the proportion of N antibody-positive individuals are provided in Supplementary Figure S4A. When we examined the influence of age on antibody responses, participants aged 79 years and older exhibited no significant longitudinal fluctuations in N-specific antibody levels, only a marginal increase was detectable between the 6- and 31-month visits (October 2020 to November 2022). Conversely, for the age groups 0–18, 19–49 and 50–79 years, notable increases in antibody levels were observed, particularly between months 12 and 26, and 26 and 31. N-specific responses gradually declined over a period of 12 months after initial infection, but significantly increased during the Omicron waves. Supplementary Figures S4B–D provide N, S IgG and S IgA antibody levels for respective age groups and statistical comparisons of all visits in each age group.

Measuring antibody isotypes separately serves to characterise immune responses further. IgA on average is produced early in immune responses while IgG appears later but lasts longer. Consistently, we observed SARS-CoV-2 S IgA antibodies decreased 6 months after infection (Figure 1D), while IgG (Figure 1C) did not significantly drop over the same period. Both IgA and IgG resurged between the 12-month (April 2021) and the 26-month mark (June 2022), a time span encompassing the Delta and Omicron BA.1 waves, and the rollout of vaccinations (before month 9). S antibodies exhibited a substantial increase (mean ratio: 7.4), surging to 1.7 times the level after infection (mean ratio: 4.4), remaining consistently high until month 31 (November 2022), with one slightly more substantial uptick between months 12 and 26 (mean IgG ratio: 7.7). This trend held true for all age groups, an overview of which is provided in Supplementary Figure S4C for IgG and Supplementary Figure S4D for IgA. In summary, S IgA antibodies decreased shortly after infection, while IgG levels remained stable, and both IgA and IgG significantly increased after COVID-19 vaccinations began.

### Cellular immune responses 

**Figure 2:**
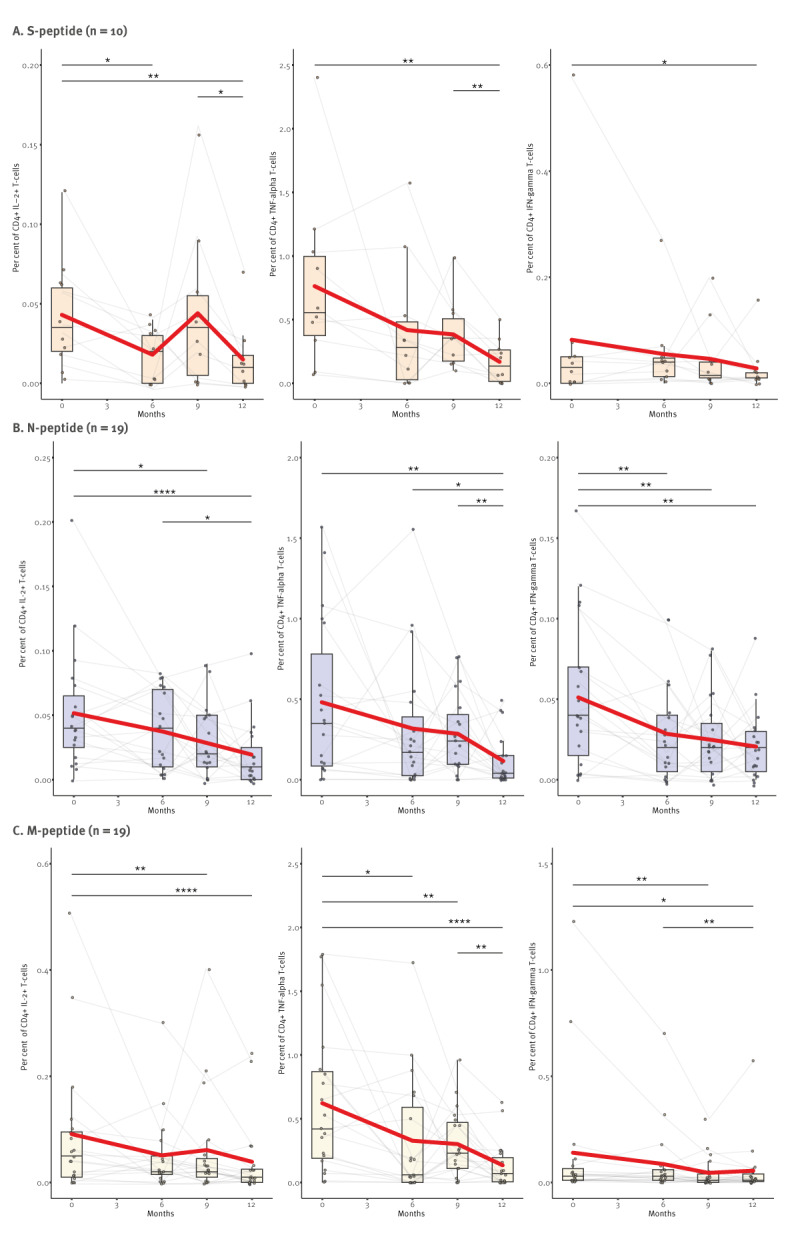
Antigen-specific CD4^+^ T-cell cytokine responses of SARS-CoV-2-infected volunteers, Heinsberg, Germany, April 2020–April 2021 (n = 19 individuals)

For a better understanding of longitudinal immune responses following SARS-CoV-2 infection, we measured the frequency of SARS-CoV-2-specific CD4^+^ T-cells expressing cytokines after restimulation with SARS-CoV-2 peptides (Figure 2). We analysed blood from 19 participants who provided samples at Visit 0 (April 2020), Visit 1 (October 2020) and Visit 3 (January 2021) (before vaccination) and who tested N antibody-positive at baseline, indicating early infection. In general, we observed a gradual reduction of cellular immune responses over time, represented by the frequency of CD4^+^ T-cells expressing IL-2, TNF-alpha or IFN-gamma, respectively. At month 9 (January 2021), there were slight upward trends in some cell types, but were not significant. The frequency of SARS-CoV-2-specific TNF-alpha^+^ CD4^+^ T-cells significantly declined for all three peptides over 12 months after infection (Figure 2A-C), but only for M did the decrease begin after 6 months (p < 0.05) (Figure 2C). In comparison, IFN-gamma-positive CD4^+^ T-cells declined over the course of 12 months (p < 0.01, p < 0.01, p < 0.0001), whereas only for N-peptides this decline already occurred after 6 months (Figure 2B, p < 0.01). The kinetics of IL-2-secreting CD4^+^ T-cells proved to be more complex, N-specific cells steadily and significantly declined between 0 and 12 months (p < 0.0001), while M- and S-specific cells increased at month 9, and declined again by month 12 (Figure 2A, significance for S: p < 0.05). Nevertheless, we did not observe significant expression differences for all three cytokines between S-, N-, and M-specific cells. When we performed a similar analysis on CD8^+^ T-cells stained for CD107a and IFN-gamma, only the frequency of M-specific CD8^+^  IFN-gamma^+^  declined over time similar to CD4^+^  cells, whereas after S and N-restimulation and for CD8^+^ CD107a^+^ cells, no significant differences were observed (Supplementary Figure S5 provides the data on IFN-gamma and CD107a expression in S, N and M peptide-restimulated CD8^+^ T cells). Collectively, these data suggest that the SARS-CoV-2-specific CD4^+^ T-cell responses were induced during SARS-CoV-2 infection, but gradually decreased 12 months after infection, while no differences in CD8^+^ T-cell responses were observed.

M: SARS-CoV-2 membrane protein; N: SARS-CoV-2 nucleocapsid protein; S: SARS-CoV-2 spike protein; SARS-CoV-2: severe acute respiratory syndrome coronavirus 2.

Frequency of cytokine-positive cells was measured via flow cytometry after SARS-CoV-2 peptide restimulation. (A) S-peptide (n = 10), (B) N-peptide (n = 19) or (C) M-peptide (n = 19) were used to stimulate PBMCs. We monitored the expression of TNF-alpha, IFN-gamma, and IL-2 by flow cytometry for different time points (0–12 months). Data are represented as individual points and box plots (interquartile range (IQR) with first quartile (Q1), median and third quartile (Q3). Minimum is defined as Q1−1.5+IQR and maximum is defined as Q3+1.5+IQR). The red line connects the median values of each time point. The Wilcoxon signed-rank tests were applied and p values < 0.05 (*), < 0.01 (**), or < 0.0001 (****) were considered to be significant.

### Seroreversion and neutralisation assessment

**Figure 3:**
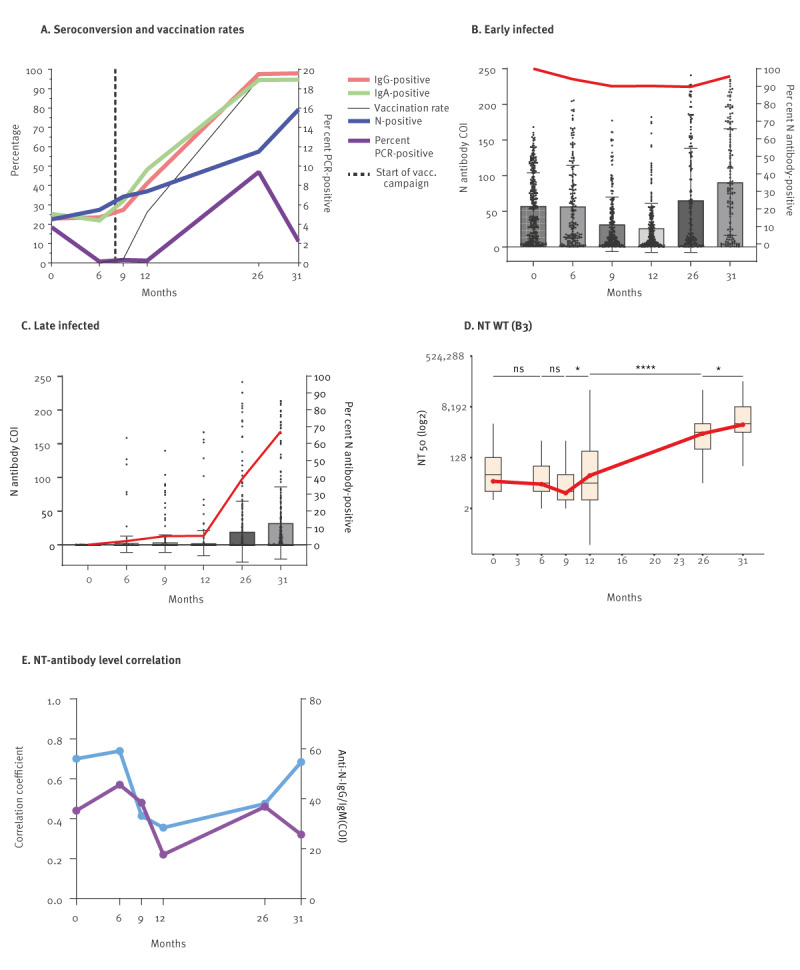
Cumulative seroconversion, acute infection and neutralisation capacity, Heinsberg, Germany, April 2020–November 2022

COI: cut-off index; IgG: immunoglobulin G; IgM immunoglobulin M; M: SARS-CoV-2 membrane protein; N: SARS-CoV-2 nucleocapsid protein; NT: neutralisation titre; S: SARS-CoV-2 spike protein; WT: wild-type.

To determine the seroprevalence over time for our entire cohort, we calculated the percentage of anti-SARS-CoV-2 N, S IgG and S IgA antibody-positive participants at the six sample collection visits. The N antibody positivity rate (Figure 3A, blue line) increased progressively from 27.4% (month 6, October 2020) to 79.3% (month 31, November 2022). The most statistically significant surge occurred between months 12 (April 2021) and 26 (June 2022), during Omicron VOC emergence (Figure 1A; p < 0.0001). Similarly, we observed a gradual rise in S-specific IgG positivity (Figure 3A, red line), starting from 23.5% at 6 months and peaking at 98.0% at 31 months, after COVID-19 vaccinations began. Based on the IgG data, we predict an average half-life of infection-induced S IgGs of 18 months. S-specific IgA exhibited a similar trend (Figure 3A, green line), ranging from 21.9% at 6 months to 94.7% at 31 months. We next determined active SARS-CoV-2 infection via RT-PCR (Supplementary Figure S1B lists the total numbers of tests performed for each visit). Acutely infected participants remained rare throughout the study (Figure 3A, purple line), fluctuating between 0.14% and 0.23% from months 6 to 12 (October 2020–April 2021). However, despite elevated antibody levels at 26 months, we noticed a rise in the number of SARS-CoV-2 PCR-positive participants (9.4%), pointing to much higher infection rates with Omicron VOC (Figure 1A).

A. The cumulative share of antibody-positive individuals given as percentage of positive S-specific IgA (green line), IgG (pink line) or N-specific ELISA (blue line). Percentage of positive PCR tests (purple) was compared with vaccination rates (black).

B–C. Comparison of immune responses of participants who were infected either at Visit 0 (April 2020) or after Visit 0 (October 2020 to November 2022). Study participants were assigned to two groups based on N antibody status at Visit 0 (April 2020): COI ≥ 1.0 (B) and COI < 1.0 (C) were analysed for antibody levels and share of positive samples. The red line shows the per cent of N antibody-positive individuals over time (right Y-axis).

D. Longitudinal neutralisation capacity. Kruskal–Wallis nonparametric tests were applied: p (9 vs 12 months) = 0.0212; p (12 vs 26 months) < 0.0001; p (26 vs 31 months) = 0.0384. The red line connects the mean values of each time point.

E. The correlation coefficient of N-specific antibody levels to neutralisation capacity (purple) compared with absolute N antibody levels (blue).

Participant numbers for respective visits are as follows: n = 726 for Visit 1; n = 1,004 for Visit 2; n = 883 for Visit 3; n = 597 for Visit 4; and n = 495 for Visit 5.

To gain insights into the nature of protection against SARS-CoV-2 reinfection, it is similarly important to determine the rate at which virus-specific antibodies disappeared, i.e. seroreversion rate, defined as COI of N-specific antibodies falling below 1.0. The noteworthy spike in positivity rate following a superspreading event meant that our cohort was well-suited to answer this question. We found that 6 months post-infection (p.i.), 5.9% of those participants infected at or before Visit 0 (April 2020) had seroreverted (Figure 3B), whereas we observed 9.9% at 9 months p.i. and 10.2% at 12 months p.i. High numbers of breakthrough infections were observed with the Delta and Omicron variants, which skewed any seroreversion analysis beyond month 12 (April 2021). Of those participants who were not infected at or before Visit 0, only 8.6% had N-antibodies by April 2021, showing low infection rates in this population before the Delta variant emerged (Figure 3B). N antibody levels in those infected after Visit 0 were also different between groups (Figure 3C, p for all time points: < 0.001). Interestingly, neutralisation capacity remained consistent between groups (data not shown).

We further evaluated the neutralisation capacity of the SARS-CoV-2 antibody-positive participants against the ancestral virus. Infections in early 2020 induced a mean neutralisation titre (NT50) of 163 (Figure 3D), which subsided in the following 9 months to 60 (36.8%). Interestingly, mean neutralisation titres against the ancestral virus were sometimes higher in the oldest group of participants (> 79 years of age) compared with the youngest (0–18 years); longitudinal neutralisation capacity by age is provided in Supplementary Figure S6. By the end of the study, the mean NT50 rose to 5,869 (Figure 3D), which is 36-fold higher than month 0, and 98-fold compared with month 9. To clarify whether N-specific antibodies exhibited increased neutralisation against VOC, we calculated the correlation coefficient between N-specific antibody levels and neutralisation over time (Figure 3E). Intriguingly, the average N-specific antibody levels displayed a similar trend as the correlation strength until month 26 but diverged between months 26 and 31 (June and November 2022).

### Next generation sequencing of early Omicron breakthrough infections

**Figure 4:**
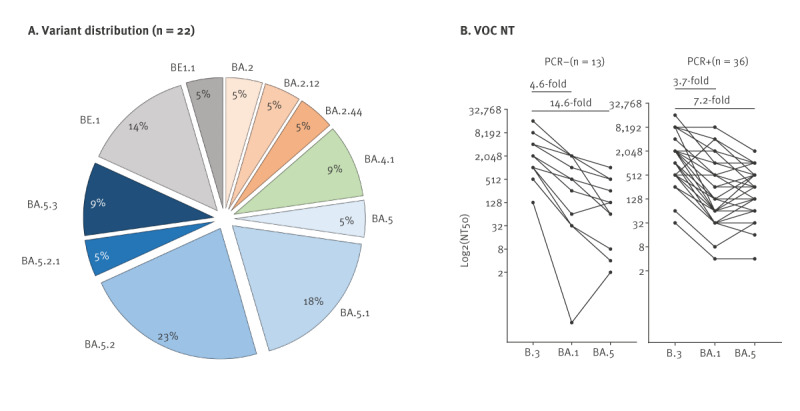
Outbreak analysis of individuals who tested SARS-CoV-2-positive during the time Omicron BA and BE variants emerged, Heinsberg, Germany, June 2022

NT: neutralisation titre; SARS-CoV-2: severe acute respiratory syndrome coronavirus 2; VOC: variant of concern.

The incidence of breakthrough infections surged with the emergence of the Omicron VOC, despite the high vaccination coverage in many countries. In our cohort, we observed high rates of Omicron infections in month 26 (June 2022), the majority of which we could identify as breakthrough infections (4/55 PCR-positive individuals were unvaccinated). When we performed NGS on 55 PCR-positive swab samples (month 26), we obtained 22 sequences, which showed a high Omicron VOC diversity (Figure 4A). Although BA.5-related variants were the most frequent, we found five different BA.5 subvariants. To assess the degree of cross-protection conferred by vaccine-induced immunity in Omicron-infected individuals, we evaluated ancestral virus and BA.1 neutralisation capacities. Although a substantial portion of the population had probably been infected by BA.1 several months earlier (it had been the dominant variant before Visit 5 (see Figure 1A), leading to a 20.5% increase in the rate of infections (Figure 3A)), the neutralisation capacity against BA.1 was notably lower than against ancestral virus by 4.6-fold for PCR-negative individuals and 3.7-fold for those acutely infected (Figure 4B). We next determined neutralisation of BA.5 and found that the difference between ancestral and Omicron variant was even greater in the PCR-negative group (14.6-fold), whereas in acutely infected individuals it was less strongly decreased (7.2-fold).

A. SARS-CoV-2 NGS of 22 of 55 participants shown as frequencies among all sequences.

B. Neutralisation capacity of plasma from sequenced samples from PCR-positive participants against the ancestral virus B.3 and the early Omicron VOC BA.1 and BA.5 compared with PCR-negative individuals. Non-neutralising samples were assigned an arbitrary value of 0.1. Due to the fact that neutralisation assays were performed with 1:2 serial dilutions of serum, the results are multiples of two and can therefore lead to overlapping lines on the graph.

### Determination of neutralisation capacities against SARS-CoV-2 variants in triple vaccinees with hybrid immunity

**Figure 5:**
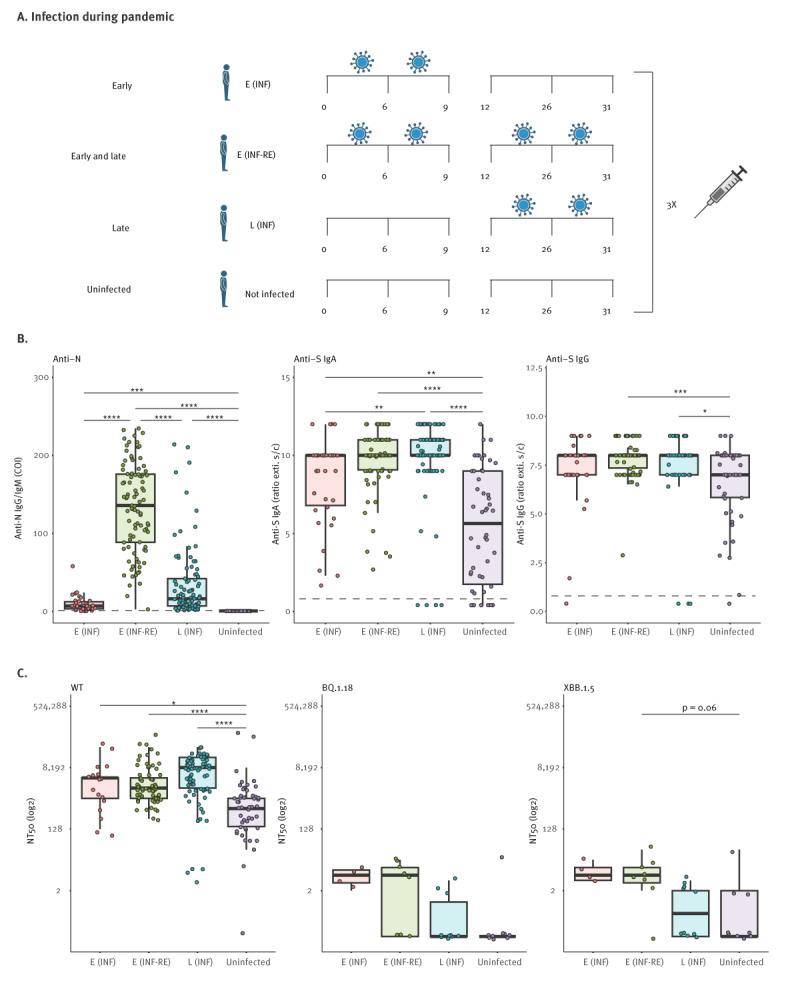
Comparative subgroup analysis of triple vaccinees, Heinsberg, Germany, April 2020–November 2022 (n = 271individuals)

To compare hybrid immunity and vaccination/infection immune responses, we categorised triple vaccinees (n = 271) into four groups, according to the time points when a rise in N-antibodies was observed (Figure 5A). Firstly, individuals who were infected during the first 9 months of the study before vaccinations were available (‘early’). Secondly, those who experienced both early SARS-CoV-2 infection and a breakthrough infection during the latter half of the study (‘early and late’). Thirdly, individuals who only had breakthrough infections in the second half of the study (‘late’). Finally, as a control group, we included triple vaccinees without infection (‘uninfected’). It is of note that participants in the ‘uninfected’ group (mean: 66 years) had a significantly higher age than those in groups ‘early and late’ (mean: 52 years; p < 0.0001) and ‘late’ (mean: 56 years, p = 0.0345). We found that the ‘early and late’ group exhibited higher N antibody spikes upon second infection compared with those infected only early or late (Figure 5B). Furthermore, breakthrough infections that occurred late resulted in more N-antibodies compared with early infections. However, infection before vaccination (‘early’) did not lead to a stronger induction of S IgGs (Figure 5B, centre). Slightly higher levels of S IgG were observed in the ‘early and late’ and ‘late’ groups compared with uninfected vaccinees. In summary, individuals who experienced both early and late infections exhibited higher N antibody levels upon second infection, while late breakthrough infections resulted in more N-antibodies compared with early infections. In terms of neutralising ancestral virus (B.3), uninfected triple vaccinees had a significantly lower capacity (mean NT50 = 3,569) than the three groups with hybrid immunity (Figure 5C). Among these three groups, the late infected participants had the highest mean neutralisation capacity (mean NT50 = 8,140) compared with those early infected (mean NT50 = 6,381) or those early AND late infected (mean NT50 = 5,923), but overall this trend was not significant.

COI: cut-off index; exti. s/c: extinction of sample divided by extinction of calibrant; IgA: immunoglobulin A; IgG: immunoglobulin G; N: SARS-CoV-2 nucleocapsid protein; S: SARS-CoV-2 spike protein; NT: neutralisation titre; WT: wild-type.

A. Group selection scheme for this analysis. Time axis is in months (0 = April 2020). Note that the two virus pictograms above each bracket stand for one possible infection, so that overall participants in one group were infected twice at the most, i.e. early and late infected were only infected once, whereas early + late were infected early and reinfected later.

B. Comparison of N and S-specific antibody levels between groups. Data are represented as individual points and box plots (interquartile range (IQR) with first quartile (Q1), median and third quartile (Q3)). Minimum is defined as Q1−1.5xIQR and maximum is defined as Q3+1.5xIQR). The dashed line represents the positivity cut-off. N numbers: early (n = 34); early and late (n = 91); late (n = 96 for N and S IgA, n = 46 for IgG); uninfected (n = 50).

C. Comparison of neutralisation capacity against ancestral virus (left), VOC BQ1.18 (centre), and VOC XBB1.5 (right). Non-neutralising samples were assigned an arbitrary value of 0.1. Kruskal–Wallis nonparametric tests were applied. P values < 0.05 (*), < 0.01 (**), < 0.001 (***) or < 0.0001 (****) were considered to be significant. N numbers for the wild-type (ancestral virus B.3) are as follows: early (n = 17); early and late (n = 65); late (n = 74); uninfected (n = 50). N numbers for BQ.1.1 and XBB.1.5: early (n = 4); early and late (n = 8); late (n = 10); uninfected (n = 9).

### Cross-neutralisation of different Omicron variants

**Figure 6:**
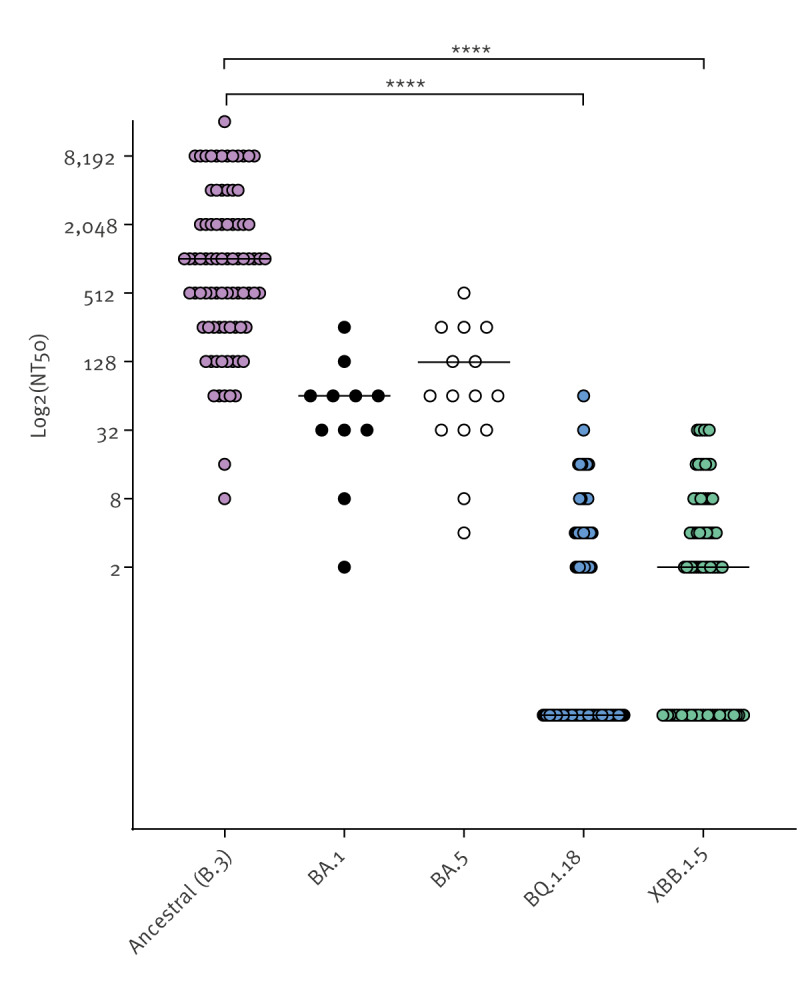
Neutralisation capacity against recent Omicron variants BQ.1.18 and XBB1.5, BA.1 and BA.5, Heinsberg, Germany, November 2021–November 2022

To examine potential disparities in immunity against various SARS-CoV-2 variants, including the ancestral virus and the Omicron subvariants BQ.1.18 and XBB1.5, we conducted an analysis of the neutralising capabilities among three distinct participant groups. These groups are: (i) individuals vaccinated four times and uninfected, (ii) individuals vaccinated three times and uninfected and (iii) individuals who were infected early in the pandemic and vaccinated three times. We found no differences between these groups in their neutralisation capacities against B3, BQ1.18 or XBB1.5, respectively. However, we found a strong reduction in neutralisation capacity against BQ.1.18 and XBB1.5 in these groups compared with ancestral virus (Figure 6), > 400-fold and > 500-fold, respectively. In most samples, which strongly neutralised ancestral virus, no neutralisation activity was observed against BQ.1.18 and XBB1.5. For a wider analysis of immune escape over time, we assessed neutralisation of the Omicron variants BA.1 and BA.5. We found the decrease of neutralisation capacity between variants to be almost gradual, although not necessarily in the order of their appearance.

Samples collected during an early Omicron wave (June 2022) were tested for neutralisation of ancestral virus (B.3), Omicron variants BA.1, BA.5, BQ.1.18 and the chimeric variant XBB.1.5. Non-neutralising samples were assigned an arbitrary value of 0.1. N numbers are: B.3 (n = 77); BA.1 (n = 11); BA.5 (n = 15); BQ.1.18 (n = 28); XBB.1.5 (n = 27).

## Discussion

The COVID-19 pandemic has been hallmarked by the rapid development of vaccines, emergence of many different SARS-CoV-2 variants, high fatality rates in elderly people and a high percentage of asymptomatic infections. In this study, we sought to further characterise both adaptive and cellular immunity during this changeable pandemic in a unique cohort. Interestingly, we observed higher N antibody levels in triple-vaccinated participants reinfected with Omicron than in triple vaccinees whose first infection was with Omicron. This indicates the existence of a diverse range of hybrid immunity profiles within the population, which varies with the timing of infections. After the first COVID-19 vaccines were introduced, we showed in a case report that the neutralising antibodies induced did not necessarily protect from infection [14], and others have shown in larger epidemiological studies that they protect from hospitalisation and death [15]. We also found that, while antibody levels did not correlate with age, the neutralisation capacity of the age group spanning 80–103 years was notably higher compared with younger age groups (specifically 0–18 and 19–49 years). Strikingly, this difference was not limited to infection, as these distinctions persisted well after vaccination. Previous studies reported a quick waning of neutralising antibodies in the age group of 70–89 years [16]. However, these disparate results might be explained by experimental differences, as Newman et al. used pseudoviruses for neutralisation. If the nature of the neutralised viral particle influences neutralisation results, this may point to the benefits of hybrid immunity and polyclonal responses in those 65 years or older, as previous studies have shown [17-19].

Throughout the SARS-CoV-2 pandemic, virus evolution interplayed in complex patterns with population immunity caused by infection, vaccination or a combination of both. Thereby, several phases emerged in the course of the pandemic, which were dominated by a single virus variant. Our study covered several of these phases; therefore, each time point is not only unique considering the spread of infection, but also in the nature of immune responses. Since the ancestral, Alpha and Delta variants infected a largely immunonaive population [20], in hindsight these early phases were marked by relatively low evolutionary pressure towards immune escape. In contrast, the first Omicron variants emerged when the population was highly vaccinated, and these variants continued to become increasingly evasive of immune response [21].

Our in-depth analysis of an Omicron outbreak in a well-characterised cohort shows high variant diversity between the two BA.2- and BA.5-dominated waves. Moreover, the PCR positivity rate at month 26 (June 2022) of the study was almost three times higher than during the original outbreak, indicating the much higher infectivity of Omicron [22], which we also confirmed via the cumulative N antibody positivity rate.

Regarding cross-protection, our findings indicate that the evolution of SARS-CoV-2 variants was not driven solely by the level of antibodies in the population, in which case cross-protection would have consistently diminished as new VOC emerged. Rather, cross-protection exhibited variability, with Omicron variants BA.1 and BA.5 displaying similarities in cross-protection despite their distinct emergence periods. This underscores the critical importance of observational studies and NGS-screening in shaping future predictive models for pandemics. Notably, later variants also induced higher N antibody responses than those earlier in the pandemic, which might be explained by mutations accumulating in N over the course of the pandemic [23]. As to the assessment of N-specific seroconversion rates as a measure of protection from severe COVID-19, it is worth considering that the population infected by Omicron variants, which led to the steepest increase in seroconversion, exhibited a decrease in hospitalisation rates and mortality [24]. This confounds the prediction of protection via antibodies from that phase of the pandemic onwards, thus limiting such projections for Omicron.

A limitation of our study is that, since some infections were inferred from a detectable N antibody response, we cannot attribute each infection to a certain variant. Yet, we argue that the strong dominance of each of the consecutive variants allowed us an informed estimate of the variant most likely causing the infection at each time point. In addition, all information we received on the vaccination status of our participants was on a voluntary basis, and therefore our dataset on the individual timing of vaccinations is incomplete. Another possible limitation concerns the ‘uninfected group’, which we defined by determining PCR, antibody and neutralisation status. We were not able to exclude participants that we determined to be uninfected who may have had contact with SARS-CoV-2 but did not mount any immune responses.

## Conclusion

Overall, longitudinal studies notably contribute to enhancing future pandemic preparedness. By documenting both the decline in neutralisation titres and the fluctuations in immune responses across different variant waves, we gained crucial insights into the dynamics of SARS-CoV-2 infection. In addition, we were able to shed light on the effect that rapid virus evolution, especially in the case of the Omicron variants, had on population immunity.

## Supplementary Data

947000 Supplement

## References

[1] Polack FP Thomas SJ Kitchin N Absalon J Gurtman A Lockhart S Safety and efficacy of the BNT162b2 mRNA Covid-19 vaccine. N Engl J Med. 2020;383(27):2603-15. 10.1056/NEJMoa2034577 33301246 PMC7745181

[2] Prasad N Bansal SB Yadav B Manhas N Yadav D Gautam S Seroconversion rate after SARS-CoV-2 infection and two doses of either ChAdOx1-nCOV COVISHIELD™ or BBV-152 COVAXIN™ vaccination in renal allograft recipients: an experience of two public and private tertiary care center. Front Immunol. 2022;13:911738. 10.3389/fimmu.2022.911738 35844596 PMC9280041

[3] Loesche M Karlson EW Talabi O Zhou G Boutin N Atchley R Longitudinal SARS-CoV-2 nucleocapsid antibody kinetics, seroreversion, and implications for seroepidemiologic studies. Emerg Infect Dis. 2022;28(9):1859-62. 10.3201/eid2809.220729 35868337 PMC9423917

[4] Guo L Wang G Wang Y Zhang Q Ren L Gu X SARS-CoV-2-specific antibody and T-cell responses 1 year after infection in people recovered from COVID-19: a longitudinal cohort study. Lancet Microbe. 2022;3(5):e348-56. 10.1016/S2666-5247(22)00036-2 35345417 PMC8942480

[5] Streeck H Schulte B Kümmerer BM Richter E Höller T Fuhrmann C Infection fatality rate of SARS-CoV2 in a super-spreading event in Germany. Nat Commun. 2020;11(1):5829. 10.1038/s41467-020-19509-y 33203887 PMC7672059

[6] Primard C Monchâtre-Leroy E Del Campo J Valsesia S Nikly E Chevandier M OVX033, a nucleocapsid-based vaccine candidate, provides broad-spectrum protection against SARS-CoV-2 variants in a hamster challenge model. Front Immunol. 2023;14:1188605. 10.3389/fimmu.2023.1188605 37409116 PMC10319154

[7] Luo M Zhou B Reddem ER Tang B Chen B Zhou R Structural insights into broadly neutralizing antibodies elicited by hybrid immunity against SARS-CoV-2. Emerg Microbes Infect. 2023;12(1):2146538. 10.1080/22221751.2022.2146538 36354024 PMC9817130

[8] Hwang S Baek SH Park D . Interaction analysis of the spike protein of Delta and Omicron variants of SARS-CoV-2 with hACE2 and Eight monoclonal antibodies using the fragment molecular orbital method. J Chem Inf Model. 2022;62(7):1771-82. 10.1021/acs.jcim.2c00100 35312321 PMC8982492

[9] Tamura T Irie T Deguchi S Yajima H Tsuda M Nasser H Virological characteristics of the SARS-CoV-2 Omicron XBB.1.5 variant. Nat Commun. 2024;15(1):1176. 10.1038/s41467-024-45274-3 38332154 PMC10853506

[10] Rössler A Netzl A Knabl L Schäfer H Wilks SH Bante D BA.2 and BA.5 omicron differ immunologically from both BA.1 omicron and pre-omicron variants. Nat Commun. 2022;13(1):7701. 10.1038/s41467-022-35312-3 36513653 PMC9745279

[11] Korencak M Sivalingam S Sahu A Dressen D Schmidt A Brand F Reconstruction of the origin of the first major SARS-CoV-2 outbreak in Germany. Comput Struct Biotechnol J. 2022;20:2292-6. 10.1016/j.csbj.2022.05.011 35574268 PMC9088089

[12] Wessendorf L Richter E Schulte B Schmithausen RM Exner M Lehmann N Dynamics, outcomes and prerequisites of the first SARS-CoV-2 superspreading event in Germany in February 2020: a cross-sectional epidemiological study. BMJ Open. 2022;12(4):e059809. 10.1136/bmjopen-2021-059809 35387836 PMC8987213

[13] Turner RJ Geraghty NJ Williams JG Ly D Brungs D Carolan MG Comparison of peripheral blood mononuclear cell isolation techniques and the impact of cryopreservation on human lymphocytes expressing CD39 and CD73. Purinergic Signal. 2020;16(3):389-401. 10.1007/s11302-020-09714-1 32754836 PMC7524993

[14] Schulte B Marx B Korencak M Emmert D Aldabbagh S Eis-Hübinger AM Case report: infection with SARS-CoV-2 in the presence of high levels of vaccine-induced neutralizing antibody responses. Front Med (Lausanne). 2021;8:704719. 10.3389/fmed.2021.704719 34368197 PMC8342944

[15] Paul P El-Naas A Hamad O Salameh MA Mhaimeed N Laswi I Effectiveness of the pre-Omicron COVID-19 vaccines against Omicron in reducing infection, hospitalization, severity, and mortality compared to Delta and other variants: A systematic review. Hum Vaccin Immunother. 2023;19(1):2167410. 10.1080/21645515.2023.2167410 36915960 PMC10054360

[16] Newman J Thakur N Peacock TP Bialy D Elrefaey AME Bogaardt C Neutralizing antibody activity against 21 SARS-CoV-2 variants in older adults vaccinated with BNT162b2. Nat Microbiol. 2022;7(8):1180-8. 10.1038/s41564-022-01163-3 35836002 PMC9352594

[17] Carazo S Skowronski DM Brisson M Sauvageau C Brousseau N Fafard J Effectiveness of previous infection-induced and vaccine-induced protection against hospitalisation due to omicron BA subvariants in older adults: a test-negative, case-control study in Quebec, Canada. Lancet Healthy Longev. 2023;4(8):e409-20. 10.1016/S2666-7568(23)00099-5 37459879

[18] Weigert M Beyerlein A Katz K Schulte R Hartl W Küchenhoff H . Vaccine-induced or hybrid immunity and COVID-19-Associated mortality during the Omicron wave. Dtsch Arztebl Int. 2023;120(13):213-20. 10.3238/arztebl.m2023.0051 37013438 PMC10277809

[19] Kim J Seo H Kim HW Kim D Kwon HJ Kim YK . Effect of Previous COVID-19 vaccination on humoral immunity 3 months after SARS-CoV-2 Omicron infection and booster effect of a fourth COVID-19 Vaccination 2 months after SARS-CoV-2 Omicron infection. Viruses. 2022;14(11):2458. 10.3390/v14112458 36366556 PMC9695529

[20] Pagani I Ghezzi S Alberti S Poli G Vicenzi E . Origin and evolution of SARS-CoV-2. Eur Phys J Plus. 2023;138(2):157. 10.1140/epjp/s13360-023-03719-6 36811098 PMC9933829

[21] Kudriavtsev AV Vakhrusheva AV Novosеletsky VN Bozdaganyan ME Shaitan KV Kirpichnikov MP Immune escape associated with RBD Omicron mutations and SARS-CoV-2 evolution dynamics. Viruses. 2022;14(8):1603. 10.3390/v14081603 35893668 PMC9394476

[22] Meng B Abdullahi A Ferreira IATM Goonawardane N Saito A Kimura I Altered TMPRSS2 usage by SARS-CoV-2 Omicron impacts infectivity and fusogenicity. Nature. 2022;603(7902):706-14. 10.1038/s41586-022-04474-x 35104837 PMC8942856

[23] Johnson BA Zhou Y Lokugamage KG Vu MN Bopp N Crocquet-Valdes PA Nucleocapsid mutations in SARS-CoV-2 augment replication and pathogenesis. PLoS Pathog. 2022;18(6):e1010627. 10.1371/journal.ppat.1010627 35728038 PMC9275689

[24] Menni C Valdes AM Polidori L Antonelli M Penamakuri S Nogal A Symptom prevalence, duration, and risk of hospital admission in individuals infected with SARS-CoV-2 during periods of omicron and delta variant dominance: a prospective observational study from the ZOE COVID Study. Lancet. 2022;399(10335):1618-24. 10.1016/S0140-6736(22)00327-0 35397851 PMC8989396

[25] Kamble P Daulatabad V Patil R John NA John J . Omicron variant in COVID-19 current pandemic: a reason for apprehension. Horm Mol Biol Clin Investig. 2022;44(1):89-96. 10.1515/hmbci-2022-0010 36064193

