# Supplementary material for: A longitudinal study on SARS-CoV-2 seroconversion, reinfection and neutralisation spanning several variant waves and vaccination campaigns, Heinsberg, Germany, April 2020 to November 2022
Source: Euro Surveill. 2024 Jun 27;29(26):2300659. doi: 10.2807/1560-7917.ES.2024.29.26.2300659 (PMC11212458; doi:10.2807/1560-7917.ES.2024.29.26.2300659)
Supplement: Supplement [file 23-00659_STREECK_Supplement.pdf]

Supplementary Figures

Disclaimer: This supplementary material is hosted by *Eurosurveillance* as supporting information alongside the article **`A Comprehensive 31-Months Longitudinal Study on SARS-CoV-2 Seroconversion, Reinfection, and Neutralization Spanning Several Variant Waves and Vaccination Campaigns´**, on behalf of the authors, who remain responsible for the accuracy and appropriateness of the content. The same standards for ethics, copyright, attributions and permissions as for the article apply. Supplements are not edited by *Eurosurveillance* and the journal is not responsible for the maintenance of any links or email addresses provided therein

Suppl. Figure S1: Longitudinal COVID-19 cohort study overview and enrollment scheme.

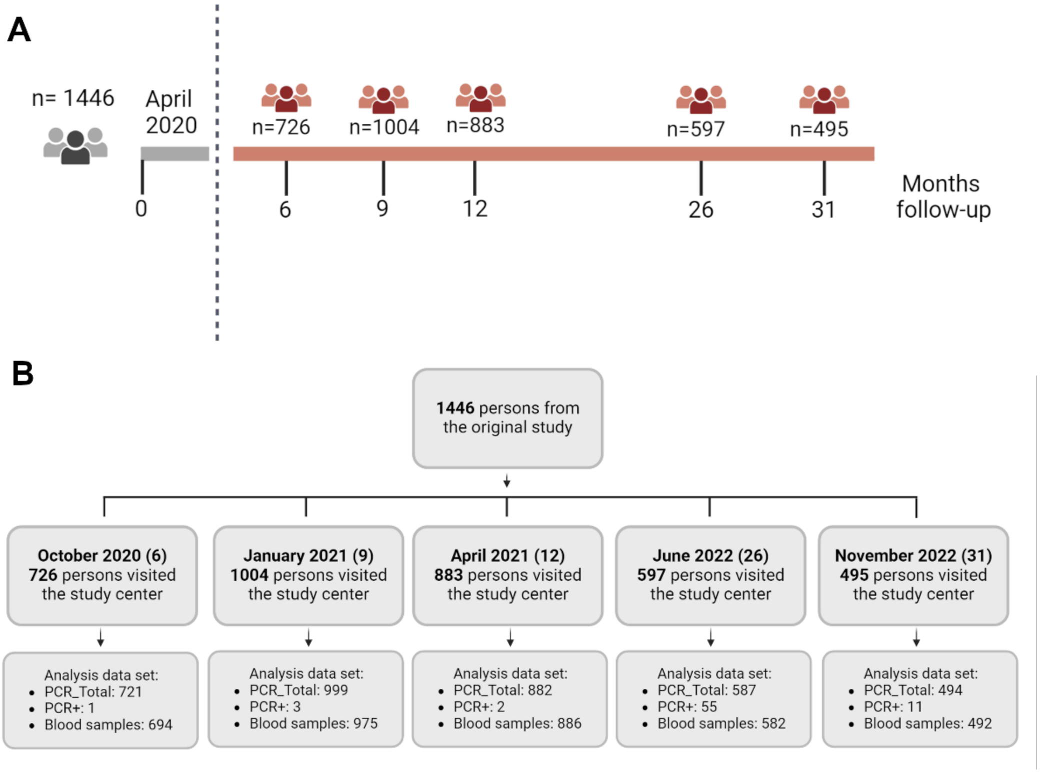

(A) Study timeline shows detailing number of participants at each visit (above) and time points of the visits (below). The grey segment represents the time point of the original COVID-19 outbreak study, which established this cohort. (B) Flow chart of enrolment beginning at the top at month 0. The numbers in brackets indicate months from the starting point of the study.

Suppl. Figure S2: Age and gender distribution of study participants.

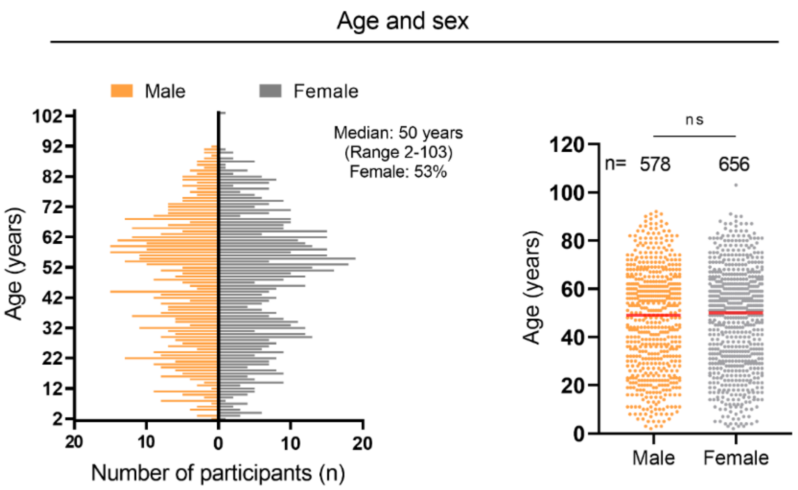

ns: not significant

The graphs show the age distribution of all participants, the distribution between female and male participants (left), as well as age distribution between female and male participants. There were no diverse participants in this study

Suppl. Figure S3: COVID-19 Incidence in Germany between March 2020 and November 2022.

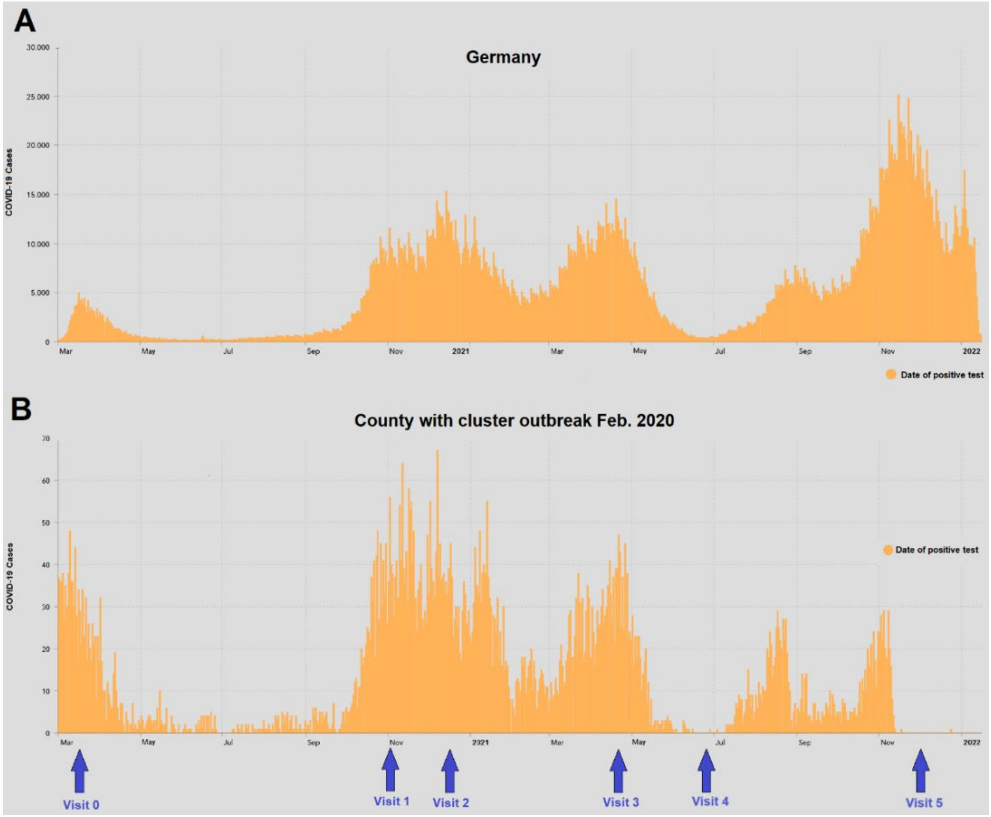

Official incidence numbers were downloaded from COVID-19 dashboard of the German federal institute for disease monitoring and prevention (Robert Koch Institute, https://covid-karte.de/). (A) Total case incidence over time for all of Germany. (B) Incidence in Heinsberg, the county which was sampled in this study. Study visits were performed starting in April 2020 (Visit 0, original cluster outbreak study), October 2020 (visit 1 of the follow-up study), January 2021 (visit 2), April 2021 (visit 3), June 2022 (visit 4), and November 2022 (visit 5).

Suppl. Figure S4: Longitudinal antibody responses of the whole cohort

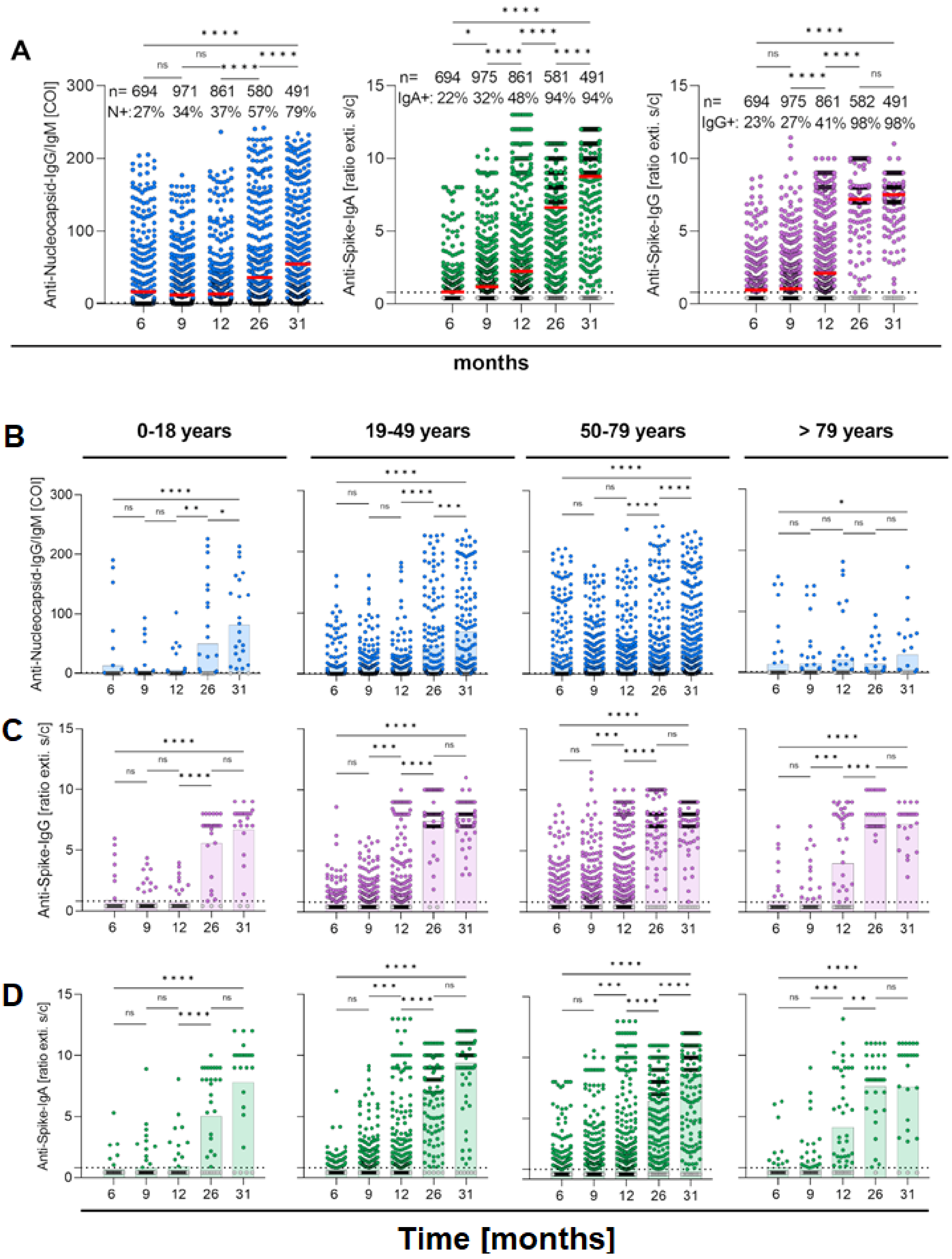

COI: cut-off index; exti. s/c: extinction of sample divided by extinction of calibrant; IgA: immunoglobulin A; IgG: immunoglobulin G; N: SARS-CoV-2 nucleocapsid protein; ns: not significant; S: SARS-CoV-2 spike protein

(A) The ELISA data from the cohort is shown as a dot scatter plot for anti-N total antibodies (blue), anti-S IgA (green), and anti-S IgG (purple). (B) With the same color-coding, antibody levels are shown divided by age groups. Note that the darker bands at certain y values do not indicate mean values, but a higher frequency of data points. Differences between the time points were assessed by the Wilcoxon signed-rank test. P values < 0.05 (*), < 0.01 (**), < 0.001 (***), or < 0.0001 (****) were considered to be significant.

Suppl. Fig. S5: Frequency analysis of antigen-specific CD8+ T cells and cytokine secretion via flow cytometry after SARS-Cov-2 peptide re-stimulation of PBMCs.

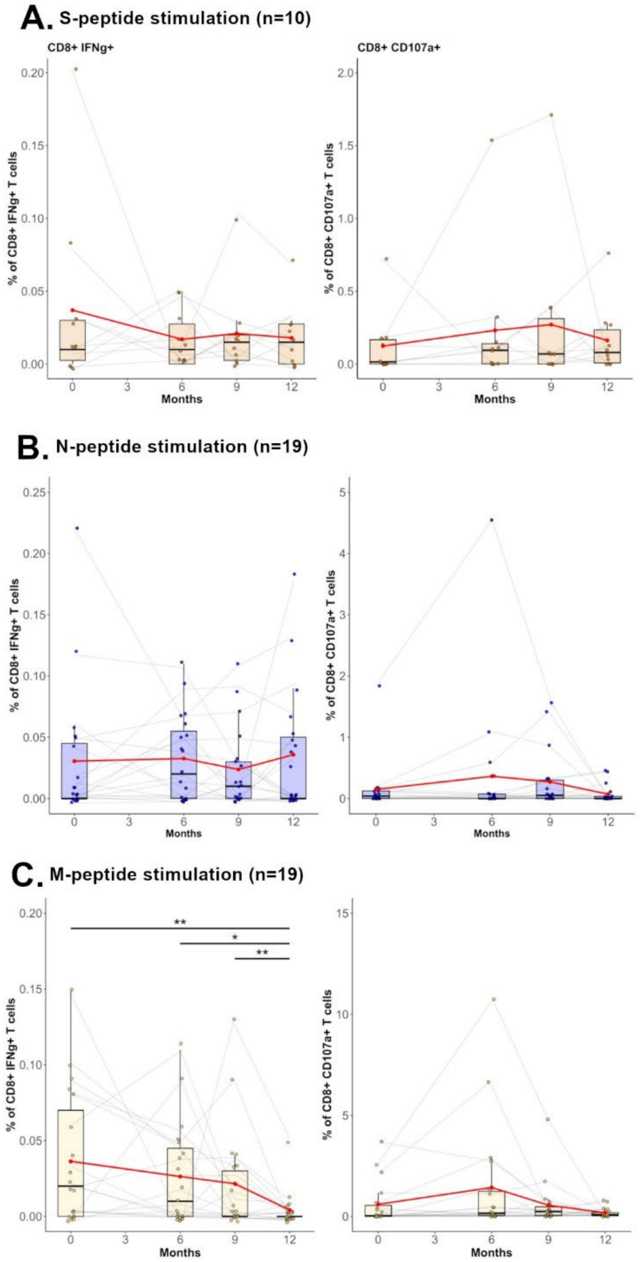

M: SARS-CoV-2 membrane protein; N: SARS-CoV-2 nucleocapsid protein; S: SARS-CoV-2 spike protein

Frequency of cytokine-positive cells was measured via flow cytometry after SARS-CoV-2 peptide re-stimulation. (A) S-peptide (n = 10), (B) N-peptide (n = 19) or (C) M-peptide (n = 19) were used to stimulate PBMCs. We monitored the expression of IFN-gamma, and CD107a from CD8 T cells by flow cytometry for different time points (0–12 months). Data are represented as individual points and box plots (interquartile range (IQR) with

first quartile (Q1), median and third quartile (Q3). Minimum is defined as Q1−1.5*IQR and maximum is defined as Q3+1.5*IQR). The red line connects the median values of each time point. The Wilcoxon signed-rank tests were applied and p values < 0.05 (*), or < 0.01 (**) were considered to be significant.

Suppl. Figure S6: Longitudinal neutralization capacity by age.

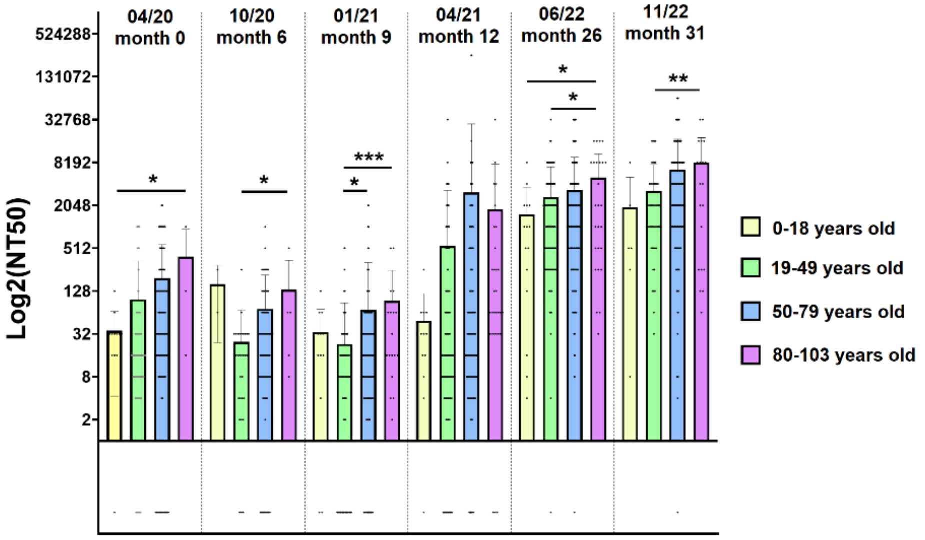

The plasma from the participants was tested for its neutralization capacity of the ancestral strain B.3. Note that the amounts of samples tested in each age group varied according to the percentage of antibody-positive participants at each time point. The individual n values for months 0/6/9/12/26/31 was 13/2/12/12/15/6 (0-18 years old), 98/32/152/158/96/52 (19-49 years old), 105/76/168/212/217/168 (50-79 years old), 3/5/18/31/26/20 (80-103 years old), respectively. The student`s t-tests were applied and p values < 0.05 (*), < 0.01 (**), or < 0.001 (***) were considered to be significant.
